# Passive limb training modulates respiratory rhythmic bursts

**DOI:** 10.1038/s41598-023-34422-2

**Published:** 2023-05-04

**Authors:** Rosamaria Apicella, Giuliano Taccola

**Affiliations:** 1 Neuroscience Department, International School for Advanced Studies (SISSA), Via Bonomea 265, Trieste, Italy; 2 Applied Neurophysiology and Neuropharmacology Lab, Istituto Di Medicina Fisica E Riabilitazione (IMFR), Via Gervasutta 48, Udine, UD Italy

**Keywords:** Neurophysiology, Respiration

## Abstract

Exercise modifies respiratory functions mainly through the afferent feedback provided by exercising limbs and the descending input from suprapontine areas, two contributions that are still underestimated in vitro. To better characterize the role of limb afferents in modulating respiration during physical activity, we designed a novel experimental in vitro platform. The whole central nervous system was isolated from neonatal rodents and kept with hindlimbs attached to an ad-hoc robot (Bipedal Induced Kinetic Exercise, BIKE) driving passive pedaling at calibrated speeds. This setting allowed extracellular recordings of a stable spontaneous respiratory rhythm for more than 4 h, from all cervical ventral roots. BIKE reversibly reduced the duration of single respiratory bursts even at lower pedaling speeds (2 Hz), though only an intense exercise (3.5 Hz) modulated the frequency of breathing. Moreover, brief sessions (5 min) of BIKE at 3.5 Hz augmented the respiratory rate of preparations with slow bursting in control (slower breathers) but did not change the speed of faster breathers. When spontaneous breathing was accelerated by high concentrations of potassium, BIKE reduced bursting frequency. Regardless of the baseline respiratory rhythm, BIKE at 3.5 Hz always decreased duration of single bursts. Surgical ablation of suprapontine structures completely prevented modulation of breathing after intense training. Albeit the variability in baseline breathing rates, intense passive cyclic movement tuned fictive respiration toward a common frequency range and shortened all respiratory events through the involvement of suprapontine areas. These observations contribute to better define how the respiratory system integrates sensory input from moving limbs during development, opening new rehabilitation perspectives.

## Introduction

Physical activity calls for an increased oxygen intake to make up for its greater demand from muscles. In turn, muscle contractions increase CO_2_ concentration in the blood stream, which is mainly sensed by baroreceptors^1^. The direct interaction between baroreceptor activation and the respiratory Central Pattern Generators (CPGs^2^) in the brainstem^3^ tunes the frequency of the respiratory rhythm. However, even before gas contents in the blood are detected, the onset of a physical activity involving the legs already generates an immediate increase in the frequency of respiration^4–8^. This phenomenon has been reported not only at the onset of volitional exercise, but also during repetitive passive leg movement in both adults and children^9^. The respiratory rhythm is composed of inspiratory and expiratory phases^10^. The former is mainly generated by two rhythmogenic oscillators, namely the parafacial respiratory group and the pre-Botzinger complex, while the expiratory rhythm arises from the Botzinger complex and the ventrotrapezoid nucleus^10,11^. In addition, respiratory circuits in the spinal cord contribute to the respiratory rhythm through the expiratory phrenic and the intercostal interneurons, as well as through spinal motoneurons in the cervical and thoraco-lumbar spinal cord driving rhythmic contractions of the diaphragm and chest muscles^10–12^.

Lumbosacral sensory afferents have a direct access to higher respiratory circuits, providing a strong functional coupling between lumbar peripheral afferents and medullary CPGs^6^. It has been widely studied how limb movement activates somatic proprioceptors that generate an afferent inflow, which entrains respiratory CPGs both via ascending long fiber tracts with paucisynaptic relays within the cord^13–15^ and through direct spinal pathways from locomotor CPGs^5,8,11,16–18^. However, activation of locomotor CPGs is not essential for respiratory rhythm entrainment in the presence of a rhythmic afferent feedback from limb motion, since the latter is per se sufficient to modulate the respiratory rhythm. For instance, manually-imposed passive movement of hindlimbs in decerebrated in vitro preparations directly increases respiratory frequency^15^. Moreover, human studies confirm that intense passive hindlimb exercise affects cardiopulmonary functions, even further when voluntary control is reduced, such as during sleep^19^.

The study aims at characterizing the purely neurogenic functional link between passive, cyclic limb movements and the respiratory rhythm.

We hypothesize that moving limbs elicit commands that ascend the spinal cord to reach the brainstem and modulate distinct features of the respiratory rhythm as a mechanism to prepare the organism for the upcoming increase in oxygen demand. Nevertheless, the involvement of a more rostral relay modulating excitability of respiratory centers should not be excluded, in line with the “*irradiation of impulses from the motor cortex*” firstly postulated by Krogh and Lindhard^4^. Afterwards, the role played by suprapontine areas in controlling respiration has been demonstrated in various conditions, including locomotion and hypoxia^11,20,21^. Whenever locomotion occurred, respiration increased at the same rate as the locomotor activity, despite any control or ablation of respiratory feedback mechanisms^20^. In addition, the level of cognitive activity alters the ventilatory response following afferent feedback from exercising limbs^22^.

The respiratory rhythm is classically studied on reduced in vitro newborn rodent preparations, either as longitudinal slices^23^ or as the en bloc brainstem^24,25^. A more intact model is represented by the brainstem + the entire spinal cord preparation, which allows to record respiratory bursts also from higher lumbar VRs^26^, although it still underestimates the modulatory influence of higher brain centers. To this regard, John Nicholls introduced the in vitro CNS preparation from young South American opossums, expressing a spontaneous regular and robust fictive respiration^27,28^. This intriguing experimental preparation has never been replicated on newborn rodents, as they were considered developmentally more mature compared to opossums and thus more prone to hypoxic damages after the long isolation procedures^29^.

The modulatory influences of suprapontine structures on spinal networks and brainstem have been recently traced in a novel newborn rat preparation that comprises the entire CNS with legs still attached^30^. The new preparation was obtained in controlled experimental conditions and allowed to record a stable respiratory rhythm for over 4 h, with a greater yield when surgical procedures were performed fast to limit any functional deficits, and when pups were younger than three days old^30^.

To explore how afferent input elicited by passive limb movement impacts on the respiratory rhythmic activity, the present study combined extracellular electrophysiological recordings of the respiratory rhythm from the whole isolated CNS preparation + legs attached with the novel robotic device named Bipedal Induced Kinetic Exercise (BIKE), which we recently created to induce rhythmic limb movement on in vitro neonatal rat preparations^31^. BIKE modulates the activity of spinal networks by passively training hindlimbs and thus generating afferent volleys which have been derived through simultaneous extracellular recordings of motor pool discharges from both dorsal roots (DRs) and ventral roots (VRs)^31^.

Then, results collected from the isolated entire CNS with legs attached have been compared to two alternative reduced preparations, represented by the traditional isolated brainstem + spinal cord^24^, and by the entire CNS after ablation of suprapontine structures.

Results from this study contribute to clarify the functional modulation provided by afferent input on respiratory circuits in response to the passive exercise of hindlimbs and the involvement of suprapontine structures in the functional neurogenic coupling between leg motion and breathing.

## Results

### A novel in vitro preparation of the entire CNS with legs attached expresses fictive respiration

To characterize the modulatory influence of suprapontine structures over the respiratory rhythm, electrical activity was continuously derived from VRs in the isolated CNS of newborn rats.

**Figure 1:**
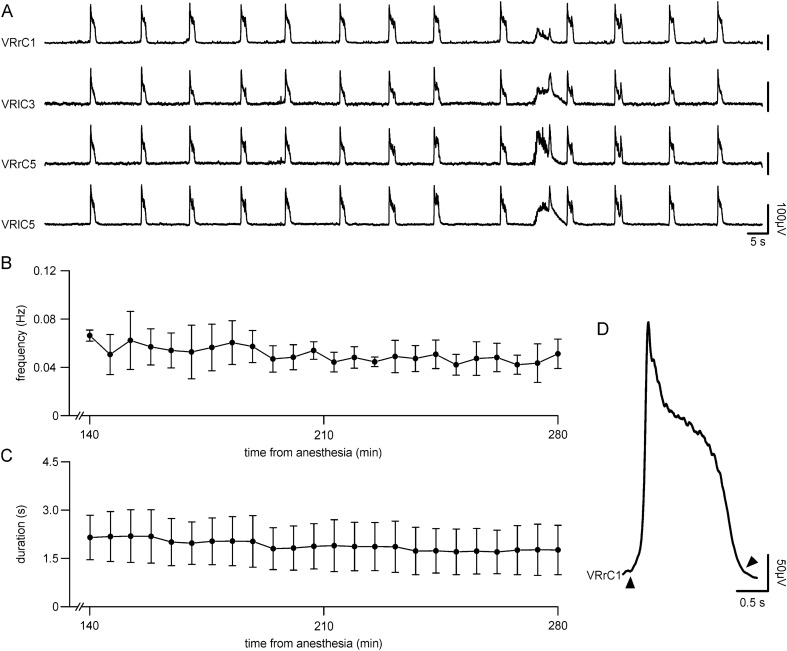
A stable fictive respiration is spontaneously recorded from cervical segments of the entire in vitro CNS with hindlimbs attached. **(A**) Simultaneous extracellular recordings acquired 2 h from the beginning of surgical isolation. Traces correspond to first and fifth cervical (rC1; rC5) ventral roots (VR) on the right side of the cord, and to the left fifth and third cervical (lC3; lC5) VRs. (**B**) Time course of the average frequency calculated for 5-min bins (black dots) showing the stability of rhythm lasting for 140 min of continuous recordings (x-axis reports the time in minutes from the induction of anesthesia at the beginning of surgical procedures for tissue isolation; n = 6). (**C**) Time course of the average burst duration calculated for 5-min bins (black dots) showing the stability of burst duration lasting for 140 min of continuous recordings (x-axis reports the time in minutes from the induction of anesthesia at the beginning of surgical procedures for tissue isolation; n = 6). (**D**) An average event out of the 40 single events from the same preparation used in A (VRrC1) showing the shape of a single respiratory burst. Note that burst duration was measured as the time distance between the onset of the trace rising from the baseline (upward arrowhead) to the point where it returns to zero (downward arrowhead).

A spontaneous respiratory rhythm was simultaneously recorded from multiple cervical VRs more than 2 h after isolation of the entire CNS with legs attached (Fig. 1A). Respiratory bursts appeared synchronous among bilateral VRs and among several cervical segments (C1-C5; Fig. 1A), as confirmed by CCF values close to 1 (CCF_rC1–rC5_ = 0.909; CCF_l-r C5_ = 0.961). Frequency of rhythm (0.07 Hz) was consistent within the range of values classically reported for preparations of the sole brainstem + spinal cord (0.1–0.2 Hz^24^). The high regularity of bursting is demonstrated by a low period CV of 15%. As a reference, stability of the respiratory rhythm period in rat pups during active sleep is between 20 and 30%^32^.

In 19 experiments, the mean rhythm frequency was 0.07 ± 0.02 Hz, with an average burst duration of 1.93 ± 0.29 s and a peak amplitude of 221.08 ± 54.66 µV.

For instance, in the same preparation used in Fig. 1A, a stable rhythm was continuously recorded until the end of the experiment, which lasted 4 h from the induction of anesthesia at the beginning of the dissecting procedures (data not shown). As demonstrated in the time course for five-minute bins in Fig. 1B, the mean rhythm frequency remained stable for at least 3 h of recordings (0.06 ± 0.01 Hz, first recording; 0.05 ± 0.01 Hz, 4 h 40 min after isolation; *P* = 0.138, paired t-test among first bins versus last bins, n = 6). Similarly, the time course for five-minute bins displays a stable mean duration of respiratory bursts for at least 3 h of recordings (Fig. 1C; 2.15 ± 0.71 s, first recording; 1.76 ± 0.77 s, 4 h 40 min after isolation; *P* = 0.899, paired t-test among first bins versus last bins, n = 6).

The seal of tight-fitting extracellular glass electrodes caused large inter-experiment variability in the amplitude of single events. Nevertheless, peak amplitude remained stable over the entire experiment, as demonstrated by similar values of mean burst amplitude at the beginning of recordings (124.95 ± 63.64 µV) and 4 h 40 min later (146.21 ± 148.68 µV; *P* = 1.000 paired t-test among first bins versus last bins, n = 6).

An average respiratory event out of a random sample of 40 individual bursts is reported in Fig. 1D. Bursts were characterized by a first peak followed by a slower decay, which returned to baseline after 1.7 s. However, double peaks occasionally occurred. In a random sample of six experiments, four of them showed scattered double bursts in the first 30 min of recordings with a mean occurrence of 22.77 ± 9.51%.

Collectively, these results point out that the whole in vitro CNS from neonatal rats is a suitable model for studying a stable respiratory rhythm for more than 4 h.

### Increasing speeds of passive pedaling distinctively affect the respiratory rhythm

**Figure 2:**
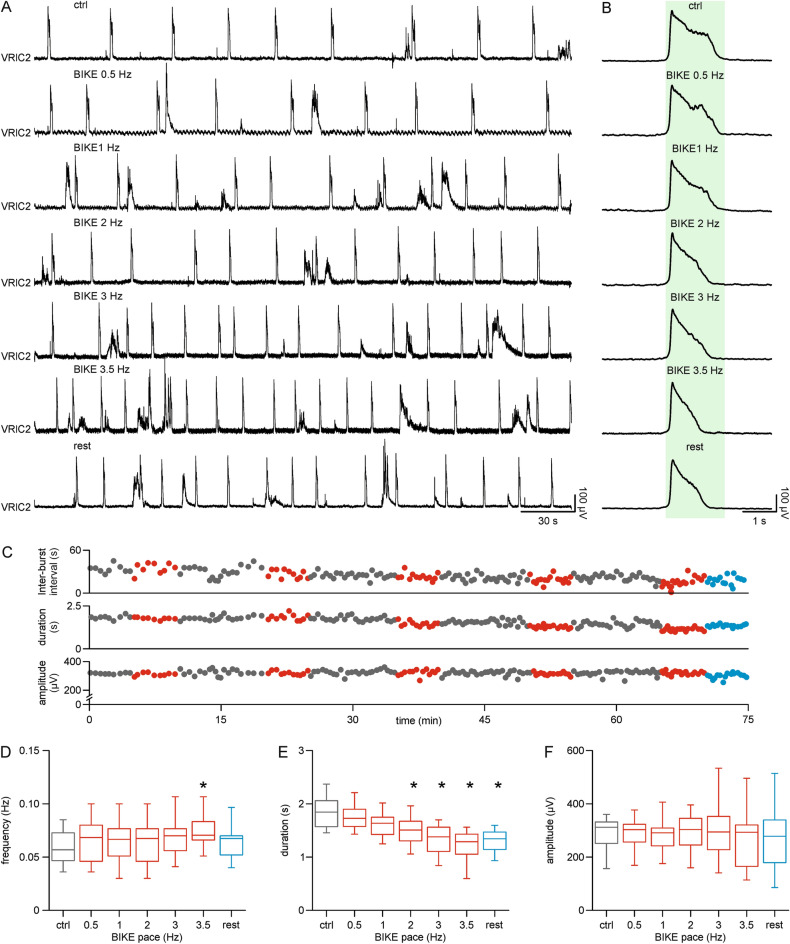
Respiratory rhythm is modulated by the progressive increase of passive pedaling speed. (**A**) 5-min traces from the right second cervical ventral root (VRlC2) in pre-BIKE control (ctrl), during passive leg mobilization at different speeds (BIKE 0.5 Hz, 1 Hz, 2 Hz, 3 Hz, 3.5 Hz) and immediately after switching off BIKE (rest). (**B)** Mean bursts of superimposed events from side-traces in (**A**). Shaded green rectangle corresponds to burst duration in control. (**C**) Time courses of inter-burst intervals, duration, and amplitude for the entire experiment related to traces in (**A**). Grey dots indicate when limbs are still, red dots correspond to steps of increasing pedaling speed and light blue dots point to the resting phase at the end of the experiment. (**D**) Whisker plot reports that respiratory frequency in control (grey box) increases during BIKE (red boxes) only at the highest speed of pedaling (3.5 Hz), recovering to baseline values right after termination of passive exercise (rest, light blue box; n = 10). (**E**) Whisker plot showing that average burst duration in control (grey box) is reduced for all BIKE speeds over 2 Hz (red boxes) without any recovery in the first post-BIKE rest (light blue box; n = 10). (**F**) Whisker plot of unchanged amplitude throughout all BIKE sessions (red boxes; n = 10). *, *P* < 0.005.

To explore whether the passive and repetitive movement of limbs affects the respiratory rhythm also through a neural mechanism entirely confined within the CNS, we created an original in vitro platform composed of the CNS from newborns rat, with hindlimbs attached to a low-noise robot (BIKE, see Supplementary video online). This preparation allows electrophysiological recordings during passive cycling at calibrated speeds^31^. Interestingly, BIKE evokes a continuous afferent volley from DRs that increases excitability of spinal networks in the cord^31^. A sample experiment is reported in Fig. 2A, where a stable respiratory rhythm is recorded from an upper cervical VR. Before turning on BIKE (ctrl), the rhythm frequency was 0.03 Hz with a duration of 1.93 s (Fig. 2B, upper trace). While the lowest pedaling speeds (0.5–1 Hz) did not affect rhythm features, the step-by-step increase in the pedaling pace (from 2 to 3.5 Hz; with a 10 min rest between each increase in the velocity of BIKE) shortened burst duration, and speeded up the respiratory rhythm only at the highest speed of exercise (3.5 Hz). At BIKE 3.5 Hz, the frequency of respiration was 0.07 Hz with burst duration equal to 1.25 s. Right after the end of serial BIKE sessions, breathing recovered towards its baseline frequency (0.05 Hz), while single events remained shorter (1.34 s). The entire time courses for inter-burst intervals, duration and amplitude show recovery to baseline values at the end of each 10 min resting period, excluding any short-term plastic events arising from the application of serial steps of increasing pedaling speed (Fig. 2C).

Mean data from 10 preparations is reported in Table 1. A reversible increase in burst frequency was displayed only at the highest intensities of training (Fig. 2D, *P* = 0.004, Friedman repeated measures ANOVA on ranks followed by multiple comparisons versus control group with Dunn's method, n = 10), whereas the duration of respiratory events was already shortened by a low BIKE pace (2 Hz), without an immediate recovery of baseline at the end of the BIKE session (Fig. 2E, *P* < 0.001, Friedman repeated measures ANOVA on ranks followed by multiple comparisons versus control group with Dunn's method, n = 10). Conversely, amplitude of bursting was not affected by exercise (Fig. 2F, *P* = 0.375, one-way repeated measures ANOVA, n = 10).

**Table 1:** Parameters of fictive respiration at increasing speeds of passive limb mobilization.

	Ctrl	BIKE 0.5 Hz	BIKE 1 Hz	BIKE 2 Hz	BIKE 3 Hz	BIKE 3.5 Hz	Rest
**Frequency (Hz)**	0.06 ± 0.02	0.07 ± 0.02	0.07 ± 0.02	0.07 ± 0.02	0.07 ± 0.02	0.08 ± 0.03	0.07 ± 0.02
**Duration (s)**	1.85 ± 0.31	1.75 ± 0.24	1.62 ± 0.23	1.50 ± 0.27	1.34 ± 0.27	1.20 ± 0.31	1.32 ± 0.22
**Amplitude (µV)**	296.65 ± 87.12	294.32 ± 80.58	298.23 ± 101.20	302.96 ± 104.86	302.86 ± 115.19	279.89 ± 119.64	276.96 ± 125.47

Repetitive passive mobilization of limbs modulated the main features of the respiratory rhythm, especially at higher training intensities. Since our model only comprises hindlimbs and the CNS, this modulatory drive must originate from the sensory afferent input evoked by pedaling, which ascends the cord to reach the supraspinal structures involved in respiration.

### Intense passive exercise differently affects breathing pace depending on baseline respiration frequency

**Figure 3:**
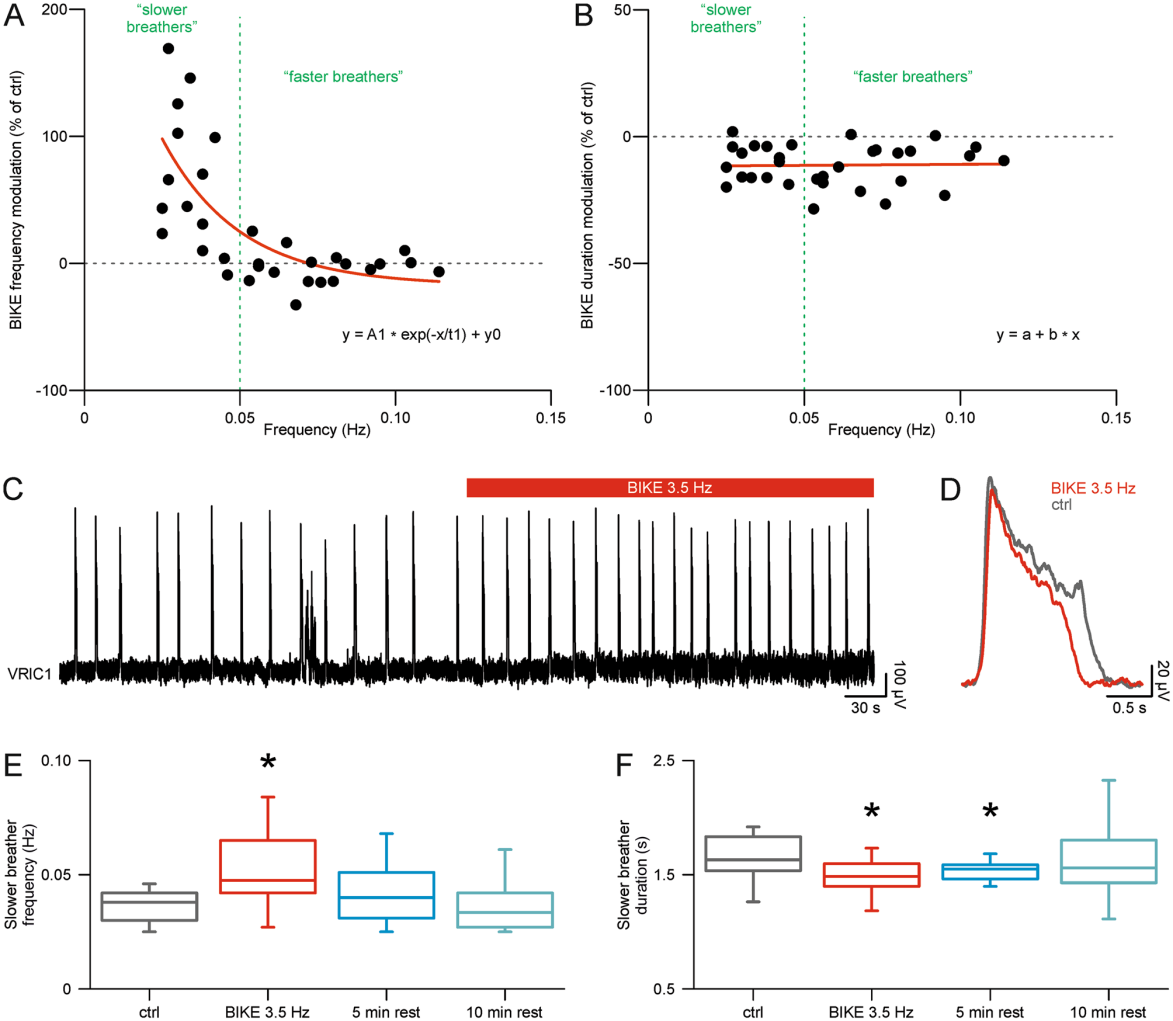
A short and intense training session differently modulates the respiratory rhythm in relation to the basal breathing pace. (**A**) Scatter plot showing on the y-axis the modulation of respiratory rhythm frequency during 5 min of BIKE at 3.5 Hz, reported as a percentage of the breathing pace in pre-BIKE control (ctrl). On the x-axis the main respiratory frequency in control is expressed in Hz. Each dot corresponds to single BIKE sessions (n = 33 preparations). The green vertical dotted line is set at a 0.05 Hz value to split the dataset in two main fields based on the speed of the respiratory rhythm in pre-BIKE control (< 0.05 Hz, indicated as “slower breathers and > 0.05 Hz, as “faster breathers”). The horizontal green dotted line traces the 0% modulation of frequency, with values above corresponding to an increase in bursting frequency and values below stating a reduction. The rhythm of *slower breathers* speeds up during BIKE, while faster breathers are mostly unaffected. Red line traces the regression curve [y = 316 (± 167) exp(− x/0.02 ± 0.01) + 83 (± 22)]. (**B**) Scatter plot illustrating that the consistent reduction of burst duration during 5 min BIKE (3.5 Hz) does not depend upon the basal breathing pace in pre-BIKE control (n = 32 preparations). Horizontal red line traces the regression line [y = 88 (± 4) + 8 (± 57) x; Pearson’s r = 0.02]. (**C**) Sample trace from a *slower breather* in control (0.04 Hz) and during the following application of BIKE (upper red bar, 5 min, 3.5 Hz) speeding up rhythm to 0.07 Hz. (**D**) Superimposed average bursts in pre-BIKE control (ctrl; grey trace) and during BIKE (3.5 Hz; red trace) obtained from the same events (ctrl 13 bursts; BIKE 18 bursts) as in the trace in (**C**). (**E**) Whisker plot of the breathing frequency for preparations identified as *slower breather* in control (grey box), summarizing the increase in rhythm frequency during BIKE (red box; 5 min; 3.5 Hz) and the following recovery of baseline values after a 5 min rest (blue box; n = 15; *, *P* = 0.001). (**F**) Whisker plot of burst duration for *slower breathers* in control (grey box), showing a reduction during BIKE (red box; 5 min; 3.5 Hz) that persists during the following resting phase (blue box) and recovers after 10 min of rest (turquoise box; n = 13; *, *P* < 0.001).

Fictive respiratory rhythm displays different periodicity in control with great variability among different samples (0.03–0.13 Hz^6,15,16,33^). To ascertain whether the modulatory effect of BIKE depends on the constitutive breathing pace of each preparation, the main frequency of fictive respiration in control is plotted on the x-axis of Fig. 3A for 33 preparations. The main frequency in control before BIKE was 0.06 ± 0.03 Hz with a wide dispersion from 0.03 Hz to 0.11 Hz. On the y-axis, the change of frequency determined by 5 min of BIKE (3.5 Hz) in each experiment is reported as a percentage of its pre-BIKE control. The horizontal dotted line divides the plot in two regions, based on the increase (above) or decrease (below) in burst frequency during BIKE. The scatter plot identifies a subgroup of preparations in which BIKE speeds up respiration (169.09 ± 54.14%). Incidentally, this breathing acceleration was reported for preparations that showed a slow respiratory rhythm in control (0.04 ± 0.01 Hz; n = 15; Fig. 3A, B, range from 0.02 to 0.05 Hz), which we called *slower breathers*. Conversely, preparations with a respiratory rhythm in control higher than 0.05 Hz (*faster breathers*; 0.07 ± 0.02 Hz; n = 18; Fig. 3A, B, range from 0.05 to 0.11 Hz) were less affected by 5 min of BIKE (97.46 ± 13.06%). Notwithstanding the breathing pace in control, 5 min BIKE always reduced burst duration to 88.75 ± 8.01% (Fig. 3B). An exemplar trace for a *slower breather* is reported before and during BIKE (3.5 Hz) in Fig. 3C, where the respiratory rhythm in control (0.04 Hz) was accelerated during five min of BIKE (0.07 Hz). Average single bursts from the same session are superimposed in Fig. 3D, where burst duration in control before BIKE (gray trace, 1.60 s) was largely decreased by passive exercise (red trace, 1.22 s). Data about changes in respiration frequency and duration of *slower breathers* are reported in Fig. 3E and F, respectively. Note the significant increase in rhythm frequency induced by BIKE 3.5 Hz (0.05 ± 0.02 Hz; Fig. 3E; *P* = 0.001; one-way repeated measures ANOVA followed by all pairwise multiple comparison procedures with Bonferroni t-test, n = 15), which recovered to pre-BIKE values after 5 min of rest (0.04 ± 0.01 Hz). In addition, regardless of the experimental protocol followed, the effect of BIKE 3.5 Hz on the rhythm pace of *slower breathers* was identical when either the pedaling speed was directly applied to untrained preparations or when 3.5 Hz was reached at the end of steps of increasing speed (*P* = 0.876; t test, n = 15–5). This observation excludes any short-term plasticity on rhythm frequency after serial BIKE sessions once at least 10 min of rest are maintained between BIKE repetitions.

Contrariwise, duration of single bursts, which was reduced by BIKE (1.68 ± 0.35 s control, 1.49 ± 0.25 s BIKE), remained shorter for even 5 min after the end of exercise (1.53 ± 0.28), but eventually recovered to baseline values after a longer rest (1.63 ± 0.33 s 10 min rest; Fig. 3F; *P* < 0.001; one-way repeated measures ANOVA followed by all pairwise multiple comparison procedures with Bonferroni t-test, n = 13).

Once the threshold of the constitutive respiratory frequency has been identified from a larger sample of animals to ascribe each preparation to either slower or faster breathers, we reconsidered the experiments with serial steps of increasing BIKE speeds (Fig. 2), reallocating the values obtained from *slower* and *faster breathers* (Supplementary Fig. S1 online). *Slower breathers* (mean rhythm frequency 0.05 ± 0.01 Hz; n = 5) showed a significant reduction in burst duration starting from 1 Hz (*P* < 0.001; one-way repeated measures ANOVA followed by multiple comparisons versus control group with Bonferroni t-test; n = 5) with an increased frequency at the highest pedaling intensity (3.5 Hz, *P* = 0.007; Friedman repeated measures ANOVA on ranks followed by multiple comparisons versus control group with Dunn's method, n = 5). Contrariwise, *faster breathers* (0.07 ± 0.01 Hz; n = 5) did not reveal any frequency changes for any of the intensities experienced (*P* = 0.178; Friedman repeated measures ANOVA on ranks followed by multiple comparisons versus control group with Dunn's method, n = 5) despite a significant reduction in burst duration starting from 1 Hz (*P* < 0.001; one-way repeated measures ANOVA followed by multiple comparisons versus control group with Bonferroni t-test; n = 5). Amplitude of respiratory events remained unchanged for both *slower* (*P* = 0.598; one-way repeated measures ANOVA, n = 5) and *faster breathers* (*P* = 0.721; one-way repeated measures ANOVA, n = 5).

These results indicate that, albeit the ample variability in baseline breathing rates, the brief (5 min) and intense (3.5 Hz) passive cyclic movement of limbs augments the respiratory rate and shortens each respiratory event in preparations displaying slow bursting in pre-BIKE control (*slower breathers*), whereas in *faster breathers* BIKE only reduces burst duration.

### Passive mobilization slows down endogenously accelerated respiratory rhythms

**Figure 4:**
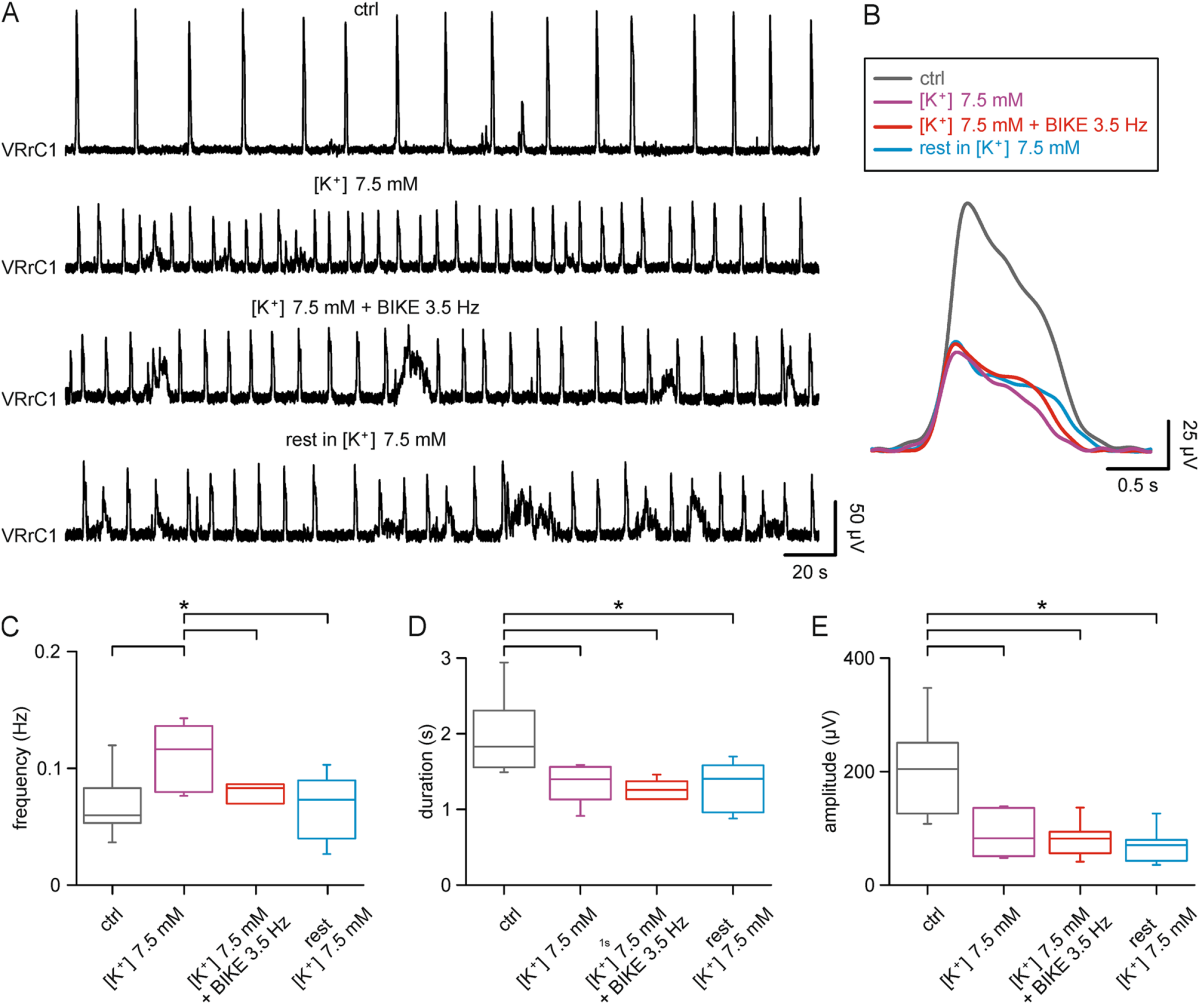
BIKE slows down the fast respiratory rhythm induced by high potassium. (**A**) Sample trace recorded from the first right ventral root (VRrC1) showing a slow respiratory rhythm in control (ctrl, 0.06 Hz), which is accelerated by perfusion in high potassium Krebs solution (7.5 mM [K^+^], 0.12 Hz), slowed down by passive pedaling in the continuous presence of high potassium (7.5 mM [K^+^] + BIKE 3.5 Hz, 0.09 Hz) and remains unchanged during rest (rest in 7.5 mM [K^+^], 0.08 Hz). (**B**) Changes in frequency are accompanied by shorter respiratory bursts (superimposed averaged single events from corresponding traces in (**A**); color code as reported in the legend above). Whisker plots summarizing changes in frequency (**C**; n = 7), burst duration (**D**; n = 7) and amplitude (**E**; n = 7) in control (ctrl, grey boxes), high potassium (7.5 mM [K^+^], purple boxes); BIKE in high potassium (7.5 mM [K^+^] + BIKE 3.5 Hz, red boxes) and rest in high potassium (rest 7.5 mM [K^+^], cyan boxes). *, P < 0.05.

To investigate any modulatory effects of passive exercise also in experimental conditions mimicking tachypnea, in a subset of four experiments, the breathing pace in control was speeded up by higher concentrations of K^+^ (7.5 mM) in the perfusing solution. As previously reported, higher doses of K^+^ augment frequency of the respiratory rhythm, while also reducing duration and peak amplitude of fictive respiratory bursts^34^. In Fig. 4A, a respiratory rhythm at 0.06 Hz, with single events that were on average 1.82 s long and 126.55 µV high (Fig. 4B), was accelerated to 0.12 Hz by 7.5 mM [K^+^]. Single bursts became shorter (1.57 s) and lower (50.89 µV) in the presence of 7.5 mM [K^+^], and were slightly reduced in duration (1.50 s), yet maintaining a similar amplitude (54.67 µV) after the following BIKE session (3.5 Hz). Remarkably, intense exercise reverted the rhythm frequency elicited by high [K^+^] toward a lower respiratory pace (0.09 Hz). After BIKE termination, the following rest in high [K^+^] showed a frequency of 0.08 Hz with bursts lasting 1.62 s while amplitude remained unchanged (54.31 µV). Data collected from seven preparations are listed in Table 2. Application of 7.5 mM [K^+^] speeded up rhythm (range from 0.08 to 0.14 Hz) and reduced both burst duration and amplitude. Intense passive exercise applied during perfusion with 7.5 mM [K^+^] decreased burst frequency, allowing recovery of the control baseline five mins after BIKE termination during rest in 7.5 mM [K^+^] (Fig. 4C; *P* < 0.05; Friedman repeated measures ANOVA on ranks followed all pairwise multiple comparison procedures with Student–Newman–Keuls Method, n = 7). Single burst duration (Fig. 4D; *P* < 0.01; one-way repeated measures ANOVA followed by all pairwise multiple comparison procedures with Bonferroni t-test, n = 7) and amplitude (Fig. 4E; *P* < 0.05; Friedman repeated measures ANOVA on ranks followed by all pairwise multiple comparison procedures with Student–Newman–Keuls Method, n = 7) were reduced in 7.5 mM [K^+^], and even further during training, displaying shorter and lower bursts than control without any recovery of values during the post-BIKE rest in 7.5 mM [K^+^]. As opposed to *slower breathers,* the respiratory rate accelerated by high [K^+^] was reduced by intense passive exercise of limbs and each respiratory event was further shortened.

**Table 2:** Changes in fictive respiration features during high potassium and BIKE (5 min).

	Ctrl rest	[K^+^] 7.5 mM	[K^+^] 7.5 mM + BIKE 3.5 Hz	[K^+^] 7.5 mM
Frequency (Hz)	0.06 ± 0.03	0.11 ± 0.03	0.08 ± 0.03	0.07 ± 0.03
Duration (s)	1.95 ± 0.52	1.34 ± 0.24	1.18 ± 0.29	1.29 ± 0.32
Amplitude (μV)	199.58 ± 84.43	85.83 ± 39.35	79.46 ± 31.77	69.99 ± 29.84

### Longer sessions of passive exercise maximize breathing modulation

**Figure 5:**
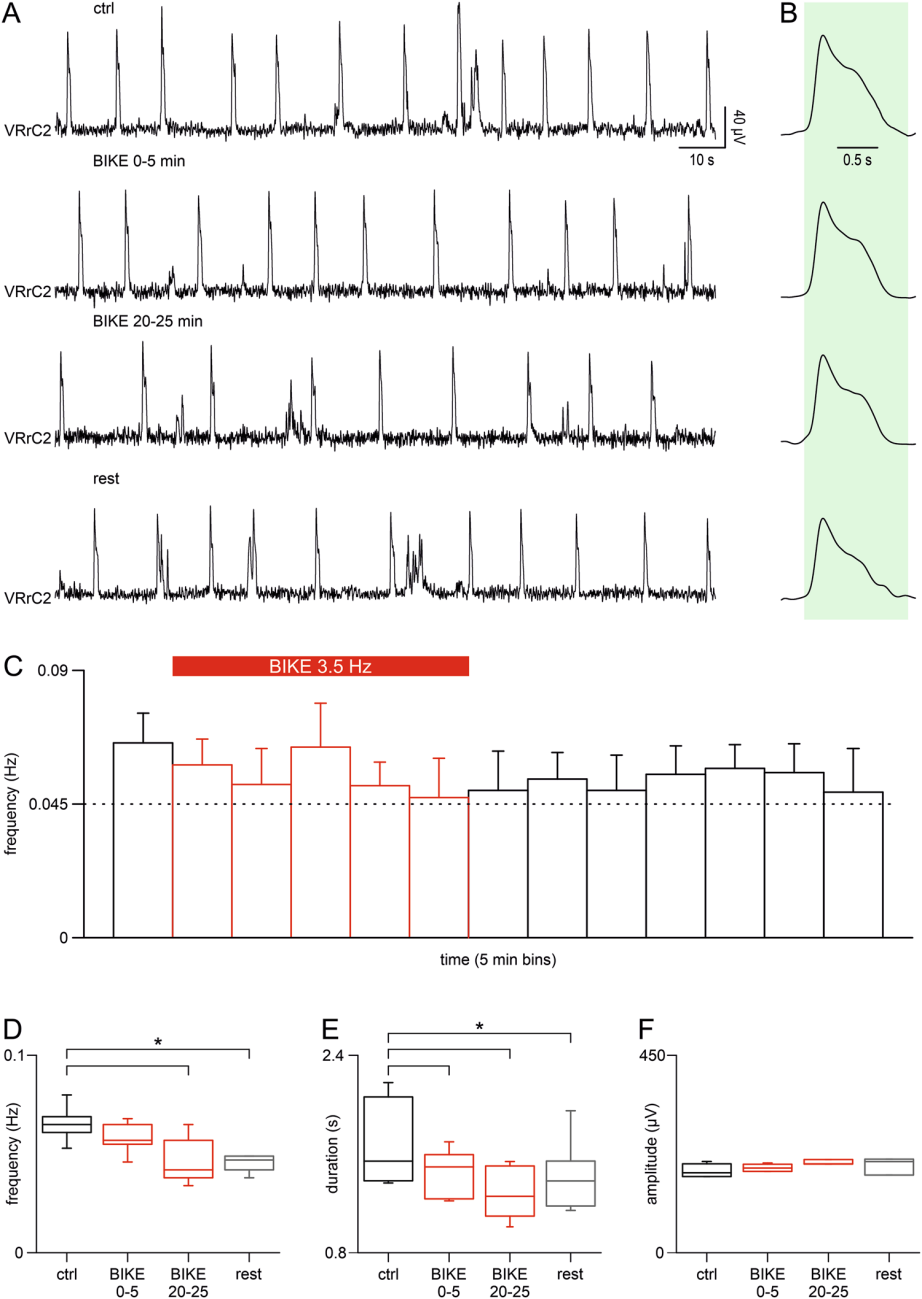
A longer BIKE session further affects the pace of bursting. (**A**) Sample traces from the right second cervical ventral root (VRrC2) recorded in pre-BIKE control (ctrl, 0.09 Hz), and during the early (BIKE 0–5 min) and late (20–25 min) phases of a long BIKE session (3.5 Hz; 25 min). (**B**) Average bursts of the corresponding traces in A showing a reduced burst duration during passive training. (**C**) Time course (bins = 5 min) tracing the dynamics of the mean rhythm frequency (Hz) from 5 preparations indicated as *faster breathers* in pre-BIKE control (0.07 ± 0.01). Passive training eventually slows down rhythm (BIKE 3.5 Hz, red bars), without recovering speed for the following 35 min of rest after the termination of pedaling. Whisker plots showing the effect of a long BIKE session as for rhythm frequency (**D**; *, *P* < 0.001; n = 5), burst duration (**E**; *, *P* < 0.001) and unchanged amplitude (**F**). Black boxes are pre-BIKE controls (ctrl), red boxes represent different phases of the BIKE training (BIKE 0–5, BIKE 20–25) and grey boxes indicate the first 5 min after BIKE termination (rest).

While intense training augments the respiratory rate of *slower breathers* in control (0.04 ± 0.01 Hz) and reduces the bursting frequency of fictive respiration when externally accelerated by high potassium (0.11 ± 0.03 Hz), the pace of most preparations showing an initial intermediate bursting rate (*faster breathers,* 0.07 ± 0.02 Hz) was not affected by a five min BIKE session. To explore whether longer sessions of passive exercise can modulate even rhythms not affected by short BIKE sessions, a 25-min training was applied to a group of five preparations with an average bursting rate in control of 0.07 ± 0.01 Hz. In Fig. 5A, a sample respiratory rhythm in control of 0.09 Hz was barely slowed down to 0.08 Hz by the first 5 min of passive limb mobilization. After 20 min of continuous pedaling, the frequency was further decreased to 0.06 Hz with a partial recovery to 0.08 Hz after turning off BIKE. In the same sample experiment, average duration of respiratory events in control (1.37 s) was reduced right after the beginning of exercise (1.22 s) and remained short until the end of pedaling (1.01 s) with a partial recovery only during post-BIKE rest (1.18 s; Fig. 5B). The time course of the mean frequency for five preparations traces the dynamics for longer BIKE sessions, indicating a rhythm deceleration (Fig. 5C). Pooled data from five experiments demonstrates that the respiratory rate, albeit unaffected by a short BIKE session, was significantly reduced by 25 min of BIKE (Fig. 5D; *P* < 0.001; one-way repeated measures ANOVA followed by multiple comparisons versus control group with Bonferroni t-test). Contrariwise, the average burst duration was equally reduced by both short and long BIKE sessions (Fig. 5E; *P* < 0.001; one-way repeated measures ANOVA followed by multiple comparisons versus control group with Bonferroni t-test), while burst amplitude remained unaffected even by longer training sessions (Fig. 5F; *P* = 0.429; one-way repeated measures ANOVA).

Collectively, data demonstrate that passive exercise always affects frequency of the respiratory rhythm, even if a distinct group of preparations (*faster breathers*) might require longer sessions to modulate the bursting rate.

### Passive training modulates respiratory frequency through a suprapontine relay

**Figure 6:**
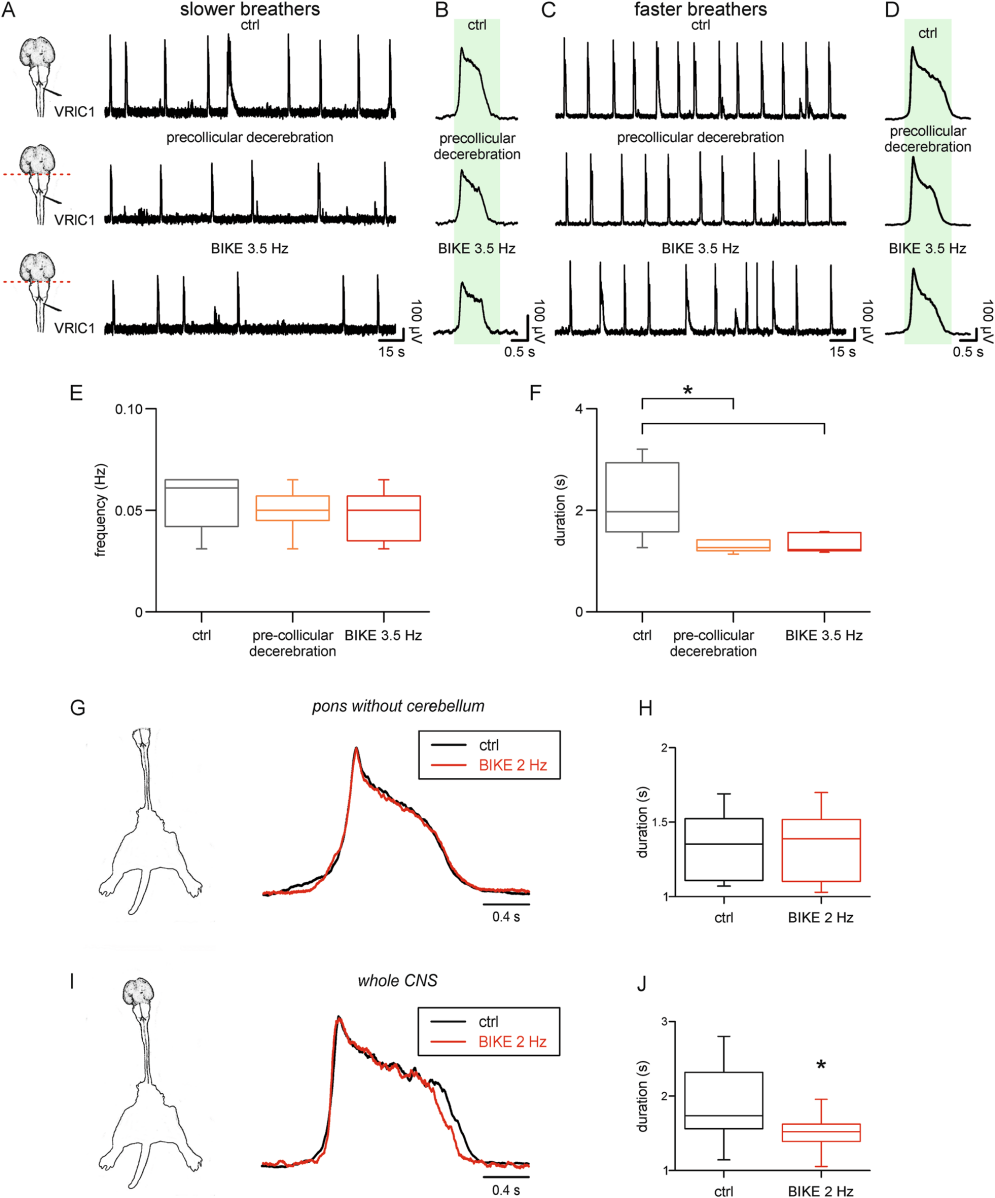
Effects of passive training on the respiratory rhythm rely on suprapontine structures. (**A**) (left). Cartoons with the ventral view of the neonatal rat brain illustrate the level of the pre-collicular transection for suprapontine decerebration (red dotted line) and the recording site from the left first cervical ventral root (VRlC1). For a *slower breather* preparation (**A**), stable fictive respiration in control (ctrl, upper trace) is slowed down after precollicular decerebration (middle trace), remaining unchanged following passive training (BIKE 3.5 Hz, 5 min). (**B**) Mean bursts from superimposed single events of the corresponding traces in A with the shaded green rectangle indicating burst duration in control. Similarly, for a *faster breather* preparation (**C**), stable fictive respiration was slowed down after precollicular decerebration and was unaffected by BIKE (3.5 Hz, 5 min). (**D**) Mean bursts from traces in C calibrated on burst duration in control (green rectangle). Whisker plots from seven experiments illustrating rhythm frequency (**E**) and burst duration (**F**; *, *P* = 0.001) in control (ctrl, grey boxes), after pre-collicular decerebration (orange boxes) and during passive training on reduced preparations (BIKE 3.5 Hz, red boxes). A short session of intense passive exercise (5 min BIKE 3.5 Hz) applied to a reduced preparation lacking suprapontine structures does not modify average rhythm frequency nor mean burst duration, as confirmed by the absence of any significant difference between orange and red boxes. (**G**) A graphical sketch of the classical preparation of the brainstem (without cerebellum) + spinal cord with legs attached. Superimposed traces correspond to identical mean respiratory bursts in control (ctrl, black trace) and during BIKE at 2 Hz (red trace). Whisker plots in (**H**) summarize mean value of burst duration before and during BIKE for 8 preparations. (**I**) Cartoon of the whole CNS with legs attached, and a pair of superimposed mean bursts in control and after BIKE 2 Hz. Note a shorter average burst during exercise (red trace). (**J**) Whisker plots reporting the statistical decrease in burst duration following passive training (*, *P* = 0.007; n = 10).

The original preparation realized for this study allows for any modulatory contributions of suprapontine structures over the in vitro respiratory rhythm. To explore whether the effects of passive training are mediated by brain centers, we applied an intense BIKE session (3.5 Hz) after a complete precollicular decerebration. In Fig. 6A, an exemplar *slower breather* preparation expresses a stable fictive respiration of 0.04 Hz in control, with single burst duration of 1.57 s (Fig. 6B, upper trace). As previously reported ^30^, surgical ablation of suprapontine structures slightly decreases rhythm frequency (0.03 Hz; Fig. 6A, middle trace) and reduces burst duration (1.31 s; Fig. 6B, middle trace). In a similar reduced preparation, which still includes the pons-medulla and the spinal cord with legs attached, application of 5 min of BIKE (3.5 Hz) did not change rhythm frequency (0.03 Hz; Fig. 6A, lower trace) nor duration of single bursts (1.32 s; Fig. 6B, lower trace) compared to pre-BIKE control. The same protocol was replicated in a prototypical *faster breather* that showed a stable fictive respiration of 0.07 Hz (Fig. 6C, upper trace), with single burst duration of 1.81 s (Fig. 6D, upper trace). Pre-collicular decerebration to ablate suprapontine centers slightly decreased rhythm frequency (0.06 Hz; Fig. 6C, middle trace) and reduced burst duration (1.42 s; Fig. 6D, middle trace). The subsequent BIKE session (5 min at 3.5 Hz) to the pons-medulla preparation did not change rhythm frequency (0.06 Hz; Fig. 6C, lower trace) nor duration of single bursts (1.44 s; Fig. 6D, lower trace) demonstrating that once suprapontine centers have been removed, BIKE did not provide any modulatory effects regardless of the constitutive respiratory rhythm (*slower* or *faster breathers*).

Similar observations were collected from seven preparations in which the surgical ablation of suprapontine structures impaired the effects of passive exercise on both the rate of respiration (Fig. 6E; *P* = 0.383; one-way repeated measures ANOVA) and its duration (Fig. 6F; *P* > 0.05; Friedman repeated measures ANOVA on ranks).

To confirm these observations, experiments were replicated in a different set of classical isolated brainstem plus spinal cord preparations ^24^ with leg attached, where BIKE was applied at 2 Hz, selected as the lowest intensity able to significantly affect burst duration (see Fig. 2E). As reported in the superimposed sample traces in Fig. 6G, average bursts were identical, showing equal duration in control (1.69 s) and after passive training (1.69 s). Pooled data from eight experiments confirms that BIKE did not affect burst duration in the isolated brainstem plus spinal cord (Fig. 6H).

Contrariwise, when the same 2 Hz training was replicated in the presence of suprapontine structures, in another group of preparations, the passive pedaling exercise shortened the duration of respiratory bursts (1.37 s) by an 11% compared to pre-BIKE control (1.56 s, Fig. 6I). Similar results were collected from ten preparations of the whole CNS, where the fresh exposure to 5 min of BIKE at 2 Hz shortened single bursts by 19 ± 12% of pre-BIKE control (Fig. 6J). These results replicate the significant reduction in burst duration due to exercise at 2 Hz that has already been reported in the experiments included in the dose–response curve of Fig. 2D (20 ± 10% of pre-BIKE control, n = 6).

Thus, the more intact preparation of the entire CNS in vitro unveils the modulatory influences of intense passive training on the respiratory rhythm.

## Discussion

In the present study, we introduced a novel in vitro experimental platform consisting in the whole CNS isolated from newborn rats, with legs attached to a mechatronic pedaling device named BIKE. The model allowed us to monitor the dynamics of the respiratory rhythm during passive exercise at calibrated speeds. In this setting, the continuous afferent volley elicited from the periphery during exercise reversibly modulated rhythmic bursts of the stable spontaneous respiratory rhythm that was extracellularly recorded synchronous for more than 4 h across all cervical ventral roots. Even short BIKE episodes at moderate speeds of pedaling always reduced the duration of respiratory events, an effect that persisted right after the end of training until eventually recovering to baseline, without affecting the amplitude of bursting. However, only an intense exercise modulated the frequency of the respiratory rhythm compared to the basal respiratory rate, with a cumulative effect appearing for longer BIKE sessions.

The novel platform allows to ascertain the pivotal contribution of suprapontine structures to the respiratory rhythm, as the ablation of suprapontine structures also abolished the modulatory effects of passive exercise on both duration and frequency of breathing.

The present study focuses on quantifying the main features of the neurogenic respiratory rhythm, while it is not intended to consider the tidal volume, which is the main descriptor of the volume of air moved into or out of the lungs during real breathing. It must be added that, especially during postnatal development, the respiratory frequency and the tidal volume change with distinct patterns^35^. Experiments with whole body plethysmography in controlled conditions of humidification and heating^35^ should be considered in the future to provide a close estimation of oxygen consumption and the resting tidal volume on fully anesthetized spontaneously breathing neonatal rats during an intense session of BIKE.

### Factors affecting duration of single respiratory bursts

In the current study, the most consistent effect of passive training on the respiratory rhythm was a robust reduction in burst duration, which appeared even at lower cycling speeds across all preparations, regardless of their basal respiratory pace. Noteworthy, our preparations showed two alternative basal paces of respiratory bursts in control: a constitutively slower pattern (*slower breathers;* respiratory frequency < 0.05 Hz) and a faster one (*faster breathers;* > 0.05 Hz), apparently uncorrelated to age, gender, or weight of animals. Although the source of this dichotomy is undetermined, a similar observation has been recently reported also in brainstem-spinal cord preparations, where slow or fast initial spontaneous respiratory rhythms were detected in control^36^. Along with the breathing pace, duration of single respiratory bursts is another main descriptor of the fictive respiration recorded from cervical VRs. While rhythm frequency corresponds to the pacemaker activity of PreBotC^37^ orchestrated by local and pontine modulatory input^37,38^, duration of single respiratory bursts in vitro is a complex and still not-fully clarified feature. In our experiments, passive limb mobilization always elicited a similar reduction in the duration of respiratory bursts, regardless of whether BIKE reduced or increased rhythm frequency. This uncoupling between changes in duration and frequency of rhythmic activities is unusual^39^ and suggests the presence of two separate pathways controlling frequency and duration of respiratory bursts. Recently, the pharmacological dissection of the two distinct cellular cascades responsible for burst duration and frequency, respectively, has been reported in neonatal rat`s transverse brainstem slices expressing a respiratory-related rhythmic motor output^40^.

In adult alive animals, the mechanism that terminates inspiration seems to be site-specific and is mainly ascribed to the ventrolateral Nucleus of the Tractus Solitarius (vlNTS) located in the brainstem. Indeed, both the cooling of neurons in the vlNTS^41^ and the silencing of neuronal activity in the NTS by lignocaine^42^ increase the duration of inspiratory bursts. In addition, microinjecting antagonists of NMDA receptors into the vlNTS produces longer inspiratory bursts, suggesting that endogenous excitatory amino acids released in the vlNTS contribute to shaping single respiratory events by acting on NMDA receptors^43^. Furthermore, selective microinjections into the vlNTS, but not into the medial NTS (mNTS), of pharmacological inhibitors of neuronal activation and/or action potential conduction increase inspiratory bursts^44^. Collectively, these observations indicate that the vlNTS region is an integral part of the so-called Inspiratory Off Switch (IOS) system required for terminating single bursts^45^. Noteworthily, respiratory neurons, localized laterally to the NTS, are in charge of switching off inspiration once lungs are inflated. These neurons project to the medulla and pons as they receive complex synaptic input from multiple local microcircuits^46,47^. As a matter of fact, retrograde labelling experiments visualized extensive projections from the retrofacial nucleus and other brainstem respiratory-related nuclei aiming at the vlNTS^48^, while axons from the vlNTS reach the Ventral Respiratory Group, including rostral inspiratory regions^49^. However, the connectome of the NTS is further crowded by the convergence of descending input. Autoradiographic experiments have shown that suprapontine excitatory input from amygdala and basal forebrain send extensive projections to the NTS, while, after horseradish peroxidase (HRP) injections to the NTS, retrogradely labeled cells have been found in the central nucleus of amygdala and in the anterior hypothalamic area^50^. The circuit described above ideally designs a direct pathway connecting distinct suprapontine structures to the autonomic regulatory nuclei in the brainstem, such as the NTS, which are possibly implicated in the regulation of respiratory bursts also when associated with emotional behaviors. For instance, electrical stimulation of a distinct area of the hypothalamus involved in defensive behaviors resulted in a short-latency excitation of a significant population of NTS interneurons that mediate the duration of hypothalamically-evoked respiratory responses^51^.

Our study provides the first description of an asymmetrical modulation induced by exercise on both, duration and frequency of the respiratory rhythm, which are also related to the intensity of exercise. While all preparations are characterized by a reduced burst duration, passive limb movement differently tunes frequency depending on the constitutive breathing pace. To the best of our knowledge, this is the first time that physical training is linked to the asymmetric modulation of the respiratory rhythm, as it has been associated in the past to distinct pharmacological manipulations^52,53^.

Our data could suggest that the respiratory frequency is not affected by limb movement when the respiratory circuit is already expressing a pace appropriate to bear the upcoming increase in oxygen demand in response to physical exercise. By merging the latter view with the results collected from ultrafast respiratory rhythms (up to 0.14 Hz) caused by high concentrations of potassium ions, we can speculate that training seems to tune the respiratory frequency around a preferred value (see in the plot of Fig. 3A the zero modulation for 0.05–0.07 Hz). In accordance with this hypothesis, BIKE facilitated slower rhythms (from 0.02 to 0.05 Hz), slightly slowed down faster breathers (from 0.05 to 0.11 Hz; actually needing 5 times longer pedaling sessions) and also decelerated artificially imposed ultrafast breathing rhythms (up to 0.14 Hz).

### Suprapontine structures involved in the modulation of the respiratory rhythm

Reminiscent of the work by Horn and Waldrop^21^ on respiratory dynamics during locomotion, we found in the isolated preparation of the entire CNS that burst duration reduced after passive exercise. Indeed, our data shows that afferent input from lumbosacral afferents reach suprapontine structures, which might represent an additional and supraspinal relay in the still undetermined ascending pathway responsible for the coupling between respiratory neural functions and dorsal afferent input during limb movement^8^.

Somatic input relaying in the first dorsal lamina of the spinal cord entrains the respiratory cycle by modulating the respiratory pattern generator both via the parabrachial region^13^ and, more directly, via several projections innervating the entire ventrolateral medulla, and in particular the retrotrapezoid nucleus (RTN). As a matter of fact, sciatic nerve electrical stimulation increases the diaphragm contractive force and frequency^54^ and facilitates neuronal firing probability in RTN, which then integrates the afferent feedback impacting on the respiratory activity during exercise^55,56^. Electrical pulses applied to the sciatic nerve increase respiratory muscle activity and breathing frequency^57^, which were however prevented by the pharmacological depression of the ventral surface of the medulla^58^. Furthermore, neuronal silencing in the parafacial region of the brainstem, including RTN, reduced the appropriate match of minute ventilation to the metabolic demands during simulated exercise involving electrical stimulation of femoral or sciatic nerves in anaesthetized rats with a spontaneous breathing^56^.

Although the neonatal rat’s neuronal networks are still immature, modulation of the respiratory rhythm is already expressed at birth, as demonstrated by characteristic changes in the frequency of fictive respiration after serially transecting the brain at various levels^30,33,38^*.* In our study, afferent input elicited by passive training failed to modulate respiratory burst features after ablating suprapontine structures. Suprapontine control of breathing has been extensively reviewed elsewhere^21,59^ showing multiple brain structures involved in modulating respiration. Noteworthy, Woldrop and collaborators described that, once receptors in muscles are activated by exercise, spinal afferents from legs, suprapontine and brainstem neurons take part in the immediate calibration of breathing at the onset of limb activity. In greater detail, they demonstrated the involvement of a specific posterior (caudal) hypothalamic area (PHA) in the control of respiration during locomotion and physical exercise. In vivo and in vitro electrophysiological recordings revealed the increased firing frequency of a large population of PHA neurons in both physically trained rats^60^ and cats^61^. In turn, electrical stimulation of the posterior hypothalamus silenced most of medullary neurons that were activated by limb exercise^62^. Exercise also increased neural transcription in numerous cardiorespiratory regions, such as the lateral and posterior hypothalamus, NTS and ventrolateral medulla (RVL)^63,64^.

Several studies confirmed the importance of the hypothalamus in modulating respiration in cats and rodents, in turn ascribing several neurogenic breathing disorders in humans to dysfunctions of the hypothalamus^59^. Further studies are needed to better identify hypothalamic neurons and their connectome, all of which are involved in the control of respiration elicited by passive limb mobilization.

In addition, since the mesencephalic periaqueductal gray (PAG) region is activated both by electrical stimulation of the hypothalamus and by exercise^65,66^, this neuronal structure is also likely responsible for the coupling of afferent input and the central command during exercise. A considerable role might also be played by the cortical respiratory motor program, as suggested by PET scan experiments in humans, showing the activation of the Supplementary Motor Area for both imagined exercises and voluntary hyperventilation^67^. Furthermore, as opposed to the classical isolated brainstem plus spinal cord model^24^, our CNS preparation always maintains the cerebellum intact. As a result, the potential role of the cerebellum in selectively modulating respiratory functions during exercise cannot be ruled out, as cerebellar neurons in the rostral fastigial nucleus respond to both passive movement and respiratory challenges^68^.

In summary, these observations demonstrate that sensory input coming from the legs are integrated at all brain levels (i.e., cortical, diencephalic, mesencephalic, cerebellar and brainstem sites), and rely on descending central commands from the posterior hypothalamus that eventually converge upon distinct rhythmogenic medullary neurons.

### Short-term plasticity of respiratory functions induced by passive training

Modulation of the ventilatory response at the onset of exercise is crucial to adequately fulfill ventilation requirements during exercise, which are further challenged by the presence of physiological (i.e. aging) and pathological conditions, or external mechanical constrains (i.e. wearing a mask^69^).

In the current study, distinct respiratory effects induced by brief and intense bouts of passive exercise, such as reduced burst duration (Figs. 2, 3, and 4), are maintained for up to 5 min after switching off BIKE, but eventually fade away to recover baseline breathing values. In a set of experiments, similar short-term changes occurred also in burst frequency (Fig. 4) compared to pre-BIKE high potassium levels, as a transient rhythm deceleration persisted even during the post-BIKE rest in high potassium.

However, long-lasting plastic changes in network activity did not occur in response to intermittent stimuli. Indeed, the respiratory baseline frequency after BIKE termination was not persistently affected by the repetition of brief (5 min) single bouts of intense training, serially (five times) supplied to the same preparation and spaced by a ten-minute rest. This is not surprising, considering that at least 70 repetitions of intense cycling were required to induce a long-term plasticity of the ventilatory response in humans^70^. Contrarywise, in our study, all changes in respiratory rhythmic bursts were abolished in less than ten minutes of rest, indicating the short functional effect of training, possibly due to the clearance of active endogenous neuromodulators released during exercise. Plasticity of ventilatory responses occurs both during development, as well as in adults^71^ following multiple interventions, such as: continuous or intermittent hypoxia^72^, dorsal rhizotomy^73^, spinal cord injury^71^, and exercise^74^. Plasticity of the ventilatory response elicited by exercise may be linked to a catecholaminergic modulation and/or serotonin (5HT)-dependent synthesis of BDNF^75^. The role of 5HT in respiratory plasticity after exercise is confirmed by the disappearance of any effect after 5-HT depletion^76^, or after the systemic^76^ or spinal^77^ pharmacological antagonism of 5-HT receptors, especially the 5-HT_2_ receptor subtype^77^.

While the exact site of action is still unknown, available evidence suggest that the critical neural mechanisms for plasticity are localized at the level of respiratory spinal motoneurons^71^. Our BIKE passive training model strengthened motoneuronal responses, as defined elsewhere by an increased amplitude of short-latency motor reflexes following DR stimulation^31^. However, in the current study, a mere potentiation of cervical spinal motoneurons would have mainly affected the amplitude of each burst, which though we recorded unchanged before and after training. Instead, short-term modulatory changes in rhythm frequency and single burst duration reported in the current study after single BIKE trials should be ascribed mainly to synapses in suprapontine regions, since these transient effects on respiratory functions were not observed in decerebrated preparations.

### Translational perspectives

Evidence about passive limb mobilization modulating respiratory functions in vitro can support the clinical translation of training-mimetic protocols to address multiple respiratory dysfunctions. In addition, passive cycling has already been applied to contrast the consequences of immobility to the musculoskeletal system, using stationary bicycles in aged persons or even in mechanically ventilated and unconscious patients in intensive care units^78^. Collectively, our results show that this minimally invasive intervention could also be extended to the early treatment of neurologically impaired subjects who are hypoventilating due to the depression of bulbospinal pathways descending toward the respiratory motor nuclei^79^.

## Methods

All procedures were approved by the International School for Advanced Studies’ (SISSA) ethics committee and are in accordance with the guidelines from the Italian Animal Welfare Act 24/3/2014 n. 26 implementing the European Union directive on animal experimentation (2010/63/ EU). The study complied with the ARRIVE guidelines.

Experiments were performed on in vitro preparations of the isolated brainstem-spinal cord and of the entire isolated CNS with hindlimbs attached, from 68 Wistar rats of either sex (0–3 day-old)^30^. Newborn rats were anesthetized by induction of hypothermia (8–9 min)^80^. This procedure allows for maintenance of all breathing functions, confirmed by the spontaneous recovery of both ventilation and heart rate, after pups’ rewarming, with the reappearance of usual cage motor behavior^81^. In hypothermic rats, once paw withdrawal reflex disappeared, the rostral part of the head above the olfactory bulbs was ablated. Skin, muscles, fatty tissues, rib cage and viscus were rapidly removed, and the preparation was then placed in a Sylgard-filled dissection chamber covered by Krebs solution bubbled with 95% O_2_–5% CO_2_ at room temperature (21–25 °C). Under microscope guidance, the skull was first opened medially, and the brain separated from the floor of the cranium, cutting all connections and cranial nerves^27^. Then, dorsal and ventral laminectomies were performed down to the lowest thoracic level (Th13), while preserving lumbo-sacral vertebrae and nerves attached to hindlimbs. Dorsal roots (DRs) and ventral roots (VRs) were kept as long as possible, removing only dorsal root ganglia (DRG). Fast surgical procedures for tissue isolation (25–40 min) allow stable recordings of spontaneous respiratory activity for at least 4 h^30^. In seven preparations, suprapontine structures were removed through surgical pre-collicular transection at the intersection between posterior cerebral and posterior communicating arteries^33,38^ to obtain a reduced preparation consisting of pons-medulla + spinal cord with legs attached. In eight preparations, the isolated brainstem + entire spinal cord was deprived of the cerebellum^24^ still keeping hindlimbs attached.

A post-dissection resting period of 30 min was systematically respected before recording procedures began. All preparations were placed and fixed with the ventral side facing up in a recording chamber (5 mL volume) continuously superfused (flow rate 7 mL/min) with oxygenated (95% O_2_–5% CO_2_) Krebs solution containing (mM): 113 NaCl, 4.5 KCl, 1 MgCl_2_7H_2_O, 2 CaCl_2_, 1 NaH_2_PO_4_, 25 NaHCO_3_ and 30 glucose, pH 7.4. In seven preparations, a modified Krebs solution with a high potassium concentration (KCl 7.5 mM) was used. Bath temperature was progressively raised to 25–27 °C and maintained for the entire duration of the experiment, as monitored with a temperature controller (TC-324C® Warner Instruments, CT, USA).

To induce passive training on the neonatal rat preparation of entire CNS with legs attached, the Bipedal Induced Kinetic Exercise (BIKE, see Supplementary video online) device designed in our laboratory^31^ was used, at different pedaling frequencies (0.5–3.5 Hz, maximum device working speed).

All recordings were taken after 60–90 min from anesthesia, as soon as a stable baseline was reached. Respiratory-like motor activities were extracellularly recorded from left (l) and right (r) cervical VRs using tight-fitting monopolar glass suction electrodes filled with Krebs solution. In experiments with suprapontine decerebration, to avoid root damage, nerves were released from electrodes before surgical transection and, afterwards, new suctions were immediately performed on the same roots. In these experiments, the two serial suctions frustrate the use of the first recordings from the same VRs as internal controls to compare the amplitude of extracellular potentials before and after transection.

DC-coupled recordings were acquired with a differential amplifier (DP-304®, Warner Instruments, CT, USA; low-pass filter = 10 kHz, high-pass filter = 0.1 Hz, gain × 1000) and analog signals further filtered by a noise eliminator (D400®, Digitimer Ltd, UK). Signals were digitized at a sampling rate of 10 kHz (Digidata 1440®, Molecular Devices Corporation, Downingtown, PA, USA), visualized real time with the software Clampex 10.7® (Molecular Devices Corporation, Downingtown, PA, USA) and stored on a PC for off-line analysis. For further calculations and figure editing, traces were processed off-line using a digital low-pass filter (10 Hz; Clampfit 10.4®, Molecular Devices Corporation, Downingtown, PA, USA).

Data analysis was performed using Clampfit 10.7® software (Molecular Devices Corporation, PA, USA) and data are expressed as mean ± SD. Cross-Correlation Function (CCF) was computed to determine the correlation among multiple cervical segments. A CCF value close to 1 at zero lag indicates that two signals are synchronous. A burst was defined as a period of sustained depolarization originating from the baseline with a rapid onset. For the selection of each event, a template of the respiratory burst waveform was generated for each animal’s VR in control conditions. The template of the designated VR for each animal was then used throughout the entire experiment to select respiratory discharges using the event detection tool of Clampfit 10.7® software (Molecular Devices Corporation, PA, USA).

Burst peak amplitude was considered as the vertical distance from the onset threshold (usually 5 times the standard deviation of baseline noise). Burst duration was defined as the time during which the baseline remained above the preset threshold^82^. Bursting frequency was obtained as the frequency components with higher magnitude in the Fast Fourier Transform (FFT).

Statistical analyses were carried out with SigmaStat® 3.5 software (Systat Software Inc, San Jose, CA, USA). All data in boxplots show the sample median (horizontal segment), 75th and 25th percentiles (top and bottom edges of box) and 1.5 times the interquartile range (whiskers). All parametric values were processed using Student’s t-test (paired) to compare two groups of data, or with a one-way repeated measures ANOVA for more than two groups. All pairwise and versus control group multiple comparisons were followed by post hoc test (Bonferroni’s t-test). Non-parametric comparisons were performed using Friedman’s repeated measures ANOVA on ranks followed by all pairwise and versus control group multiple comparison procedures with Dunn's method or by all pairwise multiple comparison procedures with Student-Newman–Keuls Method. Differences were considered statistically significant when *P* < 0.05.

### Ethics approval

The study was performed in line with the principles of the Italian Animal Welfare Act 24/3/2014 n. 26 implementing the European Union directive on animal experimentation (2010/63/ EU). The study complied with the ARRIVE guidelines.

### Consent to participate

Both authors give their formal consent to participate to the present manuscript.

## Supplementary Information

Supplementary Video 1.

Supplementary Information 1.

## Data Availability

The datasets generated during and/or analyzed during the current study are available from the corresponding author on reasonable request.

## Abbreviations

ANOVA: Analysis of variance
BIKE: Bipedal induced kinetic exercise
C: Cervical
CCF: Cross correlation function
CNS: Central nervous system
CPG: Central pattern generator
ctrl: Control
CV: Coefficient of variation
DR: Dorsal root
DRG: Dorsal root ganglia
L: Lumbar
l: Left
PAG: Periaqueductal gray
RTN: Retrotrapezoid nucleus
r: Right
VR: Ventral root

## References

[1] Forster HV, Haouzi P, Dempsey JA (2012). Control of breathing during exercise. Compr. Physiol..

[2] Sunshine MD, Sutor TW, Fox EJ, Fuller DD (2020). Targeted activation of spinal respiratory neural circuits. Exp. Neurol..

[3] Baekey DM, Molkov YI, Paton JF, Rybak IA, Dick TE (2010). Effect of baroreceptor stimulation on the respiratory pattern: Insights into respiratory-sympathetic interactions. Respir. Physiol. Neurobiol..

[4] Krogh A, Lindhard J (1913). The regulation of respiration and circulation during the initial stages of muscular work. J. Physiol..

[5] Eldridge FL, Millhorn DE, Waldrop TG (1981). Exercise hyperpnea and locomotion: Parallel activation from the hypothalamus. Science.

[6] Morin D, Viala D (2002). Coordinations of locomotor and respiratory rhythms in vitro are critically dependent on hindlimb sensory inputs. J. Neurosci..

[7] Isaev GG, Gerasimenko YP, Selionov VA, Kartashova NA (2004). Respiratory responses to voluntary and reflexly-induced stepping movements in normal subjects and spinal patients. J. Physiol. Pharmacol..

[8] Le Gal JP (2020). Modulation of respiratory network activity by forelimb and hindlimb locomotor generators. Eur. J. Neurosci..

[9] Sato Y, Katayama K, Ishida K, Miyamura M (2000). Ventilatory and circulatory responses at the onset of voluntary exercise and passive movement in children. Eur. J. Appl. Physiol..

[10] Feldman, J. L. Neurophysiology of breathing in mammals. *In Handbook of Physiology; Section 1**: The Nervous System; Volume IV: Intrinsic Regulatory Systems of the Brain.* (ed. Bloom, F. E.) 463–524 (American Physiological Society. Bethesda, MD, 1986).

[11] Shevtsova NA, Marchenko V, Bezdudnaya T (2019). Modulation of respiratory system by limb muscle afferents in intact and injured spinal cord. Front. Neurosci..

[12] Abdala AP, Rybak IA, Smith JC, Paton JF (2009). Abdominal expiratory activity in the rat brainstem-spinal cord in situ: Patterns, origins and implications for respiratory rhythm generation. J. Physiol..

[13] Potts JT, Rybak IA, Paton JF (2005). Respiratory rhythm entrainment by somatic afferent stimulation. J. Neurosci..

[14] Giraudin A, Cabirol-Pol MJ, Simmers J, Morin D (2008). Intercostal and abdominal respiratory motoneurons in the neonatal rat spinal cord: Spatiotemporal organization and responses to limb afferent stimulation. J. Neurophysiol..

[15] Giraudin A, Le Bon-Jégo M, Cabirol MJ, Simmers J, Morin D (2012). Spinal and pontine relay pathways mediating respiratory rhythm entrainment by limb proprioceptive inputs in the neonatal rat. J. Neurosci..

[16] Le Gal JP, Juvin L, Cardoit L, Thoby-Brisson M, Morin D (2014). Remote control of respiratory neural network by spinal locomotor generators. PLoS ONE.

[17] Yazawa I (2014). Reciprocal functional interactions between the brainstem and the lower spinal cord. Front. Neurosci..

[18] Le Gal JP, Juvin L, Cardoit L, Morin D (2016). Bimodal respiratory-locomotor neurons in the neonatal rat spinal cord. J. Neurosci..

[19] Loram ID, Lakie M (2002). Direct measurement of human ankle stiffness during quiet standing: The intrinsic mechanical stiffness is insufficient for stability. J. Physiol..

[20] Eldridge FL, Millhorn DE, Kiley JP, Waldrop TG (1985). Stimulation by central command of locomotion, respiration and circulation during exercise. Respir. Physiol..

[21] Horn EM, Waldrop TG (1998). Suprapontine control of respiration. Respir. Physiol..

[22] Bell HJ, Duffin J (2004). Respiratory response to passive limb movement is suppressed by a cognitive task. J. Appl. Physiol..

[23] Johnson SM, Smith JC, Funk GD, Feldman JL (1994). Pacemaker behavior of respiratory neurons in medullary slices from neonatal rat. J. Neurophysiol..

[24] Suzue T (1984). Respiratory rhythm generation in the in vitro brain stem-spinal cord preparation of the neonatal rat. J. Physiol..

[25] Onimaru H (1995). Studies of the respiratory center using isolated brainstem-spinal cord preparations. Neurosci. Res..

[26] Dubayle D, Viala D (1996). Interactions between medullary and spinal respiratory rhythm generators in the in vitro brainstem spinal cord preparation from newborn rats. Exp. Brain. Res..

[27] Nicholls JG, Stewart RR, Erulkar SD, Saunders NR (1990). Reflexes, fictive respiration and cell division in the brain and spinal cord of the newborn opossum, *Monodelphis domestica*, isolated and maintained in vitro. J. Exp. Biol..

[28] Eugenín J, Nicholls JG (2000). Control of respiration in the isolated central nervous system of the neonatal opossum, *Monodelphis domestica*. Brain. Res. Bull..

[29] Wilson RJ, Chersa T, Whelan PJ (2003). Tissue PO2 and the effects of hypoxia on the generation of locomotor-like activity in the in vitro spinal cord of the neonatal mouse. Neuroscience.

[30] Mohammadshirazi, A., Apicella, R., Zylberberg, B. A., Mazzone, G. L. & Taccola, G. Suprapontine structures modulate brainstem and spinal networks. *Cell. Mol. Neurobiol.*10.1007/s10571-023-01321-z (2023). 10.1007/s10571-023-01321-z PMC10333404 36732488

[31] Dingu N, Deumens R, Taccola G (2018). Afferent input induced by rhythmic limb movement modulates spinal neuronal circuits in an innovative robotic in vitro preparation. Neuroscience.

[32] Davis MR, Magnusson JL, Cummings KJ (2019). Increased central cholinergic drive contributes to the apneas of serotonin-deficient rat pups during active sleep. J. Appl. Physiol..

[33] Voituron N, Frugière A, Gros F, Macron JM, Bodineau L (2005). Diencephalic and mesencephalic influences on ponto-medullary respiratory control in normoxic and hypoxic conditions: An in vitro study on central nervous system preparations from newborn rat. Neuroscience.

[34] Rybak IA, Shevtsova NA, St-John WM, Paton JF, Pierrefiche O (2003). Endogenous rhythm generation in the pre-Bötzinger complex and ionic currents: modelling and in vitro studies. Eur. J. Neurosci..

[35] Julien C, Bairam A, Joseph V (2008). Chronic intermittent hypoxia reduces ventilatory long-term facilitation and enhances apnea frequency in newborn rats. Am. J. Physiol. Regul. Integr. Comp. Physiol..

[36] Nicolosi A (2018). Acute exposure to zinc oxide nanoparticles critically disrupts operation of the respiratory neural network in neonatal rat. Neurotoxicology.

[37] Yang CF, Kim EJ, Callaway EM, Feldman JL (2020). Monosynaptic projections to excitatory and inhibitory preBötzinger complex neurons. Front. Neuroanat..

[38] Okada Y, Kawai A, Mückenhoff K, Scheid P (1998). Role of the pons in hypoxic respiratory depression in the neonatal rat. Respir. Physiol..

[39] Bracci E, Beato M, Nistri A (1997). Afferent inputs modulate the activity of a rhythmic burst generator in the rat disinhibited spinal cord in vitro. J. Neurophysiol..

[40] Morgado-Valle C, Smith JC, Fernandez-Ruiz J, Lopez-Meraz L, Beltran-Parrazal L (2023). Modulation of inspiratory burst duration and frequency by bombesin in vitro. Pflugers Arch..

[41] Koepchen HP, Abel HH, Klüssendorf D (1987). Integrative neurovegetative and motor control: Phenomena and theory. Funct. Neurol..

[42] Budzińska K, Romaniuk JR (1995). The role of raphe and tractus solitarius neuronal structures in the modulation of respiratory pattern in rabbits. Acta Neurobiol. Exp. (Wars).

[43] Berger I (1995). NMDA receptors are involved at the ventrolateral nucleus tractus solitarii for termination of inspiration. Eur. J. Pharmacol..

[44] Wasserman AM, Sahibzada N, Hernandez YM, Gillis RA (2000). Specific subnuclei of the nucleus tractus solitarius play a role in determining the duration of inspiration in the rat. Brain Res..

[45] Poon CS, Song G (2014). Bidirectional plasticity of pontine pneumotaxic postinspiratory drive: Implication for a pontomedullary respiratory central pattern generator. Prog. Brain Res..

[46] Miyazaki M, Arata A, Tanaka I, Ezure K (1998). Activity of rat pump neurons is modulated with central respiratory rhythm. Neurosci. Lett..

[47] Miyazaki M, Tanaka I, Ezure K (1999). Excitatory and inhibitory synaptic inputs shape the discharge pattern of pump neurons of the nucleus tractus solitarii in the rat. Exp. Brain Res..

[48] Portillo F, Pásaro R (1987). Axonal projections to the ventrolateral nucleus of the solitary tract revealed by double labelling of retrograde fluorescent markers in the cat. Neurosci. Lett..

[49] Núñez-Abades PA, Morillo AM, Pásaro R (1993). Brainstem connections of the rat ventral respiratory subgroups: Afferent projections. J. Auton. Nerv. Syst..

[50] Schwaber JS, Kapp BS, Higgins GA, Rapp PR (1982). Amygdaloid and basal forebrain direct connections with the nucleus of the solitary tract and the dorsal motor nucleus. J. Neurosci..

[51] Silva-Carvalho L, Dawid-Milner MS, Spyer KM (1995). The pattern of excitatory inputs to the nucleus tractus solitarii evoked on stimulation in the hypothalamic defence area in the cat. J. Physiol..

[52] Del Negro CA, Kam K, Hayes JA, Feldman JL (2009). Asymmetric control of inspiratory and expiratory phases by excitability in the respiratory network of neonatal mice in vitro. J. Physiol..

[53] Feldman JL, Del Negro CA, Gray PA (2013). Understanding the rhythm of breathing: So near, yet so far. Annu. Rev. Physiol..

[54] Schiefer M, Gamble J, Strohl KP (2018). Sciatic nerve stimulation and its effects on upper airway resistance in the anesthetized rabbit model relevant to sleep apnea. J. Appl. Physiol..

[55] Kanbar R, Stornetta RL, Guyenet PG (2016). Sciatic nerve stimulation activates the retrotrapezoid nucleus in anesthetized rats. J. Neurophysiol..

[56] Korsak A, Sheikhbahaei S, Machhada A, Gourine AV, Huckstepp RTR (2018). The role of parafacial neurons in the control of breathing during exercise. Sci. Rep..

[57] Haxhiu MA, van Lunteren E, Mitra J, Cherniack NS, Strohl KP (1984). Comparison of the responses of the diaphragm and upper airway muscles to central stimulation of the sciatic nerve. Respir. Physiol..

[58] Strohl KP (1988). Nasal and tracheal responses to chemical and somatic afferent stimulation in anesthetized cats. J. Appl. Physiol..

[59] Fukushi I, Yokota S, Okada Y (2019). The role of the hypothalamus in modulation of respiration. Respir. Physiol. Neurobiol..

[60] Beatty JA, Kramer JM, Plowey ED, Waldrop TG (2005). Physical exercise decreases neuronal activity in the posterior hypothalamic area of spontaneously hypertensive rats. J. Appl. Physiol..

[61] Waldrop TG, Stremel RW (1989). Muscular contraction stimulates posterior hypothalamic neurons. Am. J. Physiol..

[62] Nolán PC, Waldrop TG (1997). Integrative role of medullary neurons of the cat during exercise. Exp. Physiol..

[63] Iwamoto GA, Wappel SM, Fox GM, Buetow KA, Waldrop TG (1996). Identification of diencephalic and brainstem cardiorespiratory areas activated during exercise. Brain Res..

[64] Ichiyama RM, Gilbert AB, Waldrop TG, Iwamoto GA (2002). Changes in the exercise activation of diencephalic and brainstem cardiorespiratory areas after training. Brain Res..

[65] Kramer JM, Waldrop TG (1998). Neural control of the cardiovascular system during exercise. An integrative role for the vestibular system. J. Vestib. Res..

[66] Basnayake S (2011). Identifying cardiovascular neurocircuitry involved in the exercise pressor reflex in humans using functional neurosurgery. J. Appl. Physiol..

[67] Thornton JM (2001). Identification of higher brain centres that may encode the cardiorespiratory response to exercise in humans. J. Physiol..

[68] Lutherer LO, Williams JL, Everse SJ (1989). Neurons of the rostral fastigial nucleus are responsive to cardiovascular and respiratory challenges. J. Auton. Nerv. Syst..

[69] Babb TG, Wood HE, Mitchell GS (2010). Short- and long-term modulation of the exercise ventilatory response. Med. Sci. Sports Exerc..

[70] Wood HE, Fatemian M, Robbins PA (2003). A learned component of the ventilatory response to exercise in man. J. Physiol..

[71] Mitchell GS, Johnson SM (2003). Neuroplasticity in respiratory motor control. J. Appl. Physiol..

[72] Mitchell GS (2001). Invited review: Intermittent hypoxia and respiratory plasticity. J. Appl. Physiol..

[73] Mitchell GS, Sloan HE, Foley KT, Brownfield MS, Miletic V (1992). Increased serotonin in the thoracic spinal cord of goats following chronic thoracic dorsal rhizotomy (TDR). FASEB J..

[74] Martin PA, Mitchell GS (1993). Long-term modulation of the exercise ventilatory response in goats. J. Physiol..

[75] Baker-Herman TL (2004). BDNF is necessary and sufficient for spinal respiratory plasticity following intermittent hypoxia. Nat. Neurosci..

[76] Babb TG (1997). Ventilation and respiratory mechanics during exercise in younger subjects breathing CO2 or HeO2. Respir. Physiol..

[77] Mitchell GS, Turner DL, Henderson DR, Foley KT (2008). Spinal serotonin receptor activation modulates the exercise ventilatory response with increased dead space in goats. Respir. Physiol. Neurobiol..

[78] Camargo Pires-Neto R (2013). Very early passive cycling exercise in mechanically ventilated critically ill patients: Physiological and safety aspects-a case series. PLoS ONE.

[79] Malone IG, Nosacka RL, Nash MA, Otto KJ, Dale EA (2021). Electrical epidural stimulation of the cervical spinal cord: Implications for spinal respiratory neuroplasticity after spinal cord injury. J. Neurophysiol..

[80] Danneman PJ, Mandrell TD (1997). Evaluation of five agents/methods for anesthesia of neonatal rats. Lab. Anim. Sci..

[81] Zimmer MB, Fong AY, Milsom WK (2020). Effect of temperature, age and the pons on respiratory rhythm in the rat brainstem-spinal cord. Respir. Physiol. Neurobiol..

[82] Bracci E, Ballerini L, Nistri A (1996). Spontaneous rhythmic bursts induced by pharmacological block of inhibition in lumbar motoneurons of the neonatal rat spinal cord. J. Neurophysiol..

