# Supplementary figures and images for: Passive limb training modulates respiratory rhythmic bursts
Source: Sci Rep. 2023 May 4;13:7226. doi: 10.1038/s41598-023-34422-2 (PMC10160044; doi:10.1038/s41598-023-34422-2)

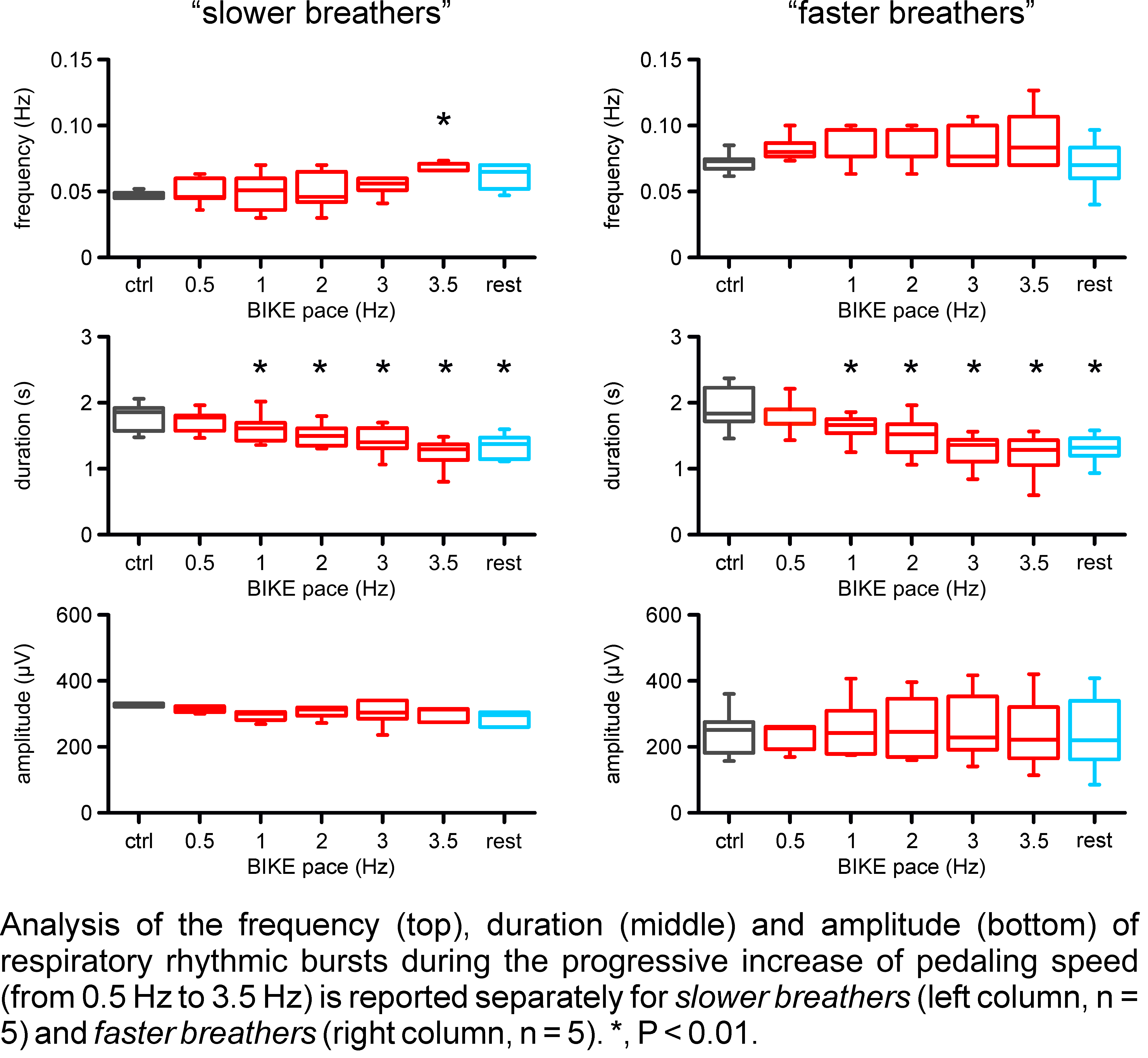

Supplement: Supplementary file 2 — Supplementary Information 1. [file 41598_2023_34422_MOESM2_ESM.tif]
